# Jurors’ perceptions of transgender victims of sexual assault: A literature review of empirical research and policy review of judicial instructions

**DOI:** 10.1002/bsl.2694

**Published:** 2024-08-28

**Authors:** Gianni Ribeiro, Faye T. Nitschke

**Affiliations:** ^1^ School of Law and Justice University of Southern Queensland Ipswich QLD Australia; ^2^ School of Psychological Sciences University of Newcastle Callaghan NSW Australia

**Keywords:** judicial instructions, juror decision‐making, rape myths, sexual assault, transgender, victim stereotype

## Abstract

Sexual assault affects many people of all gender identities, yet most cases do not result in conviction. This may be due to common, inaccurate misperceptions juries hold about how sexual assault is perpetrated and how victims respond to sexual assault. Research has examined misperceptions relating to cisgender victims, yet little is known about the unique misconceptions and stereotypes that may unfairly disadvantage transgender victims or whether courts are attempting to safeguard against them. This article presents a literature review of empirical research on (mock) jurors’ perceptions of transgender victims and a review of judicial instructions about gender identity. We find that empirical research is extremely limited with mixed findings, but many jurisdictions allow for judicial instructions warning jurors against prejudice based on gender identity. Further research is urgently needed to identify common misperceptions jurors may have that are specific to transgender victims to inform legal safeguards and improve justice outcomes.

## 1 INTRODUCTION

Sexual assault^1^ is a global concern, with transgender^2^ individuals experiencing disproportionately higher rates of victimisation (Callander et al., 2019; James et al., 2016). However, there is significant attrition of sexual assault cases within the criminal justice system, ultimately resulting in most reported cases failing to reach a conviction (Daly & Bouhours, 2010). This may be due, in part, to common (but inaccurate) stereotypes, beliefs, and biases about sexual assault which can influence jury decision‐making (e.g., Bohner & Schapansky, 2018; Süssenbach, 2016). While existing research has explored these misperceptions concerning cisgender victims,^3^ scant attention has been paid to the unique biases decision‐makers may hold about transgender victims and whether legal mechanisms adequately address these to ensure decision‐making is accurate. As such, this article bridges this gap by reviewing empirical research on (mock) jurors’ perceptions of transgender victims and examining existing judicial instructions regarding gender identity.

### 1.1 Prevalence of sexual assault and attrition of cases through the criminal justice system

Sexual assault is a deeply concerning global issue, with approximately 30% of women worldwide having endured sexual assault since the age of 15 (World Health Organization, 2021). In Australia, 22% of women and 6.1% of men have experienced sexual assault since the age of 15 (Australian Bureau of Statistics, 2023), with comparable rates seen in the United States of America (hereafter, USA; Smith et al., 2018), Canada (Cotter & Savage, 2019), and New Zealand (New Zealand Ministry of Justice, 2022). It is important to note that prevalence research has largely focussed on the gender binary, reporting prevalence rates for individuals who identify as a man or woman without any further information regarding sex assigned at birth or gender identity (Fileborn, 2012). However, the limited available research indicates that transgender individuals face disproportionately high rates of sexual assault victimisation of 47%–50% (Callander et al., 2019; James et al., 2016)—placing them at over 4 times the risk compared to cisgender individuals (Flores et al., 2021).

Despite these alarming prevalence rates, most sexual assaults do not progress through the criminal justice system. The highest attrition occurs at the reporting stage, as the majority of sexual assaults are not formally reported to police. In Australia, only 8.3% of women who had been sexually assaulted by men reported the incident to police (Australian Bureau of Statistics, 2023). Reporting rates are similarly low in Canada (6%; Cotter, 2021) and New Zealand (6%; New Zealand Ministry of Justice, 2022), though somewhat higher in the USA at 21.4% (U.S. Department of Justice, 2023). Furthermore, transgender individuals exhibit some of the lowest reporting rates among sexual assault victims, with as few as 6% reporting to law enforcement agencies in the USA (Langenderfer‐Magruder et al., 2016).

Even when sexual assaults are reported to police, most cases never progress to prosecution, let alone conviction. In Australia, only 6.9% of reported sexual assaults result in the perpetrator being charged and just 5.7% reach the court stage (Cox., 2016). International analysis reveals similar trends; of all reported cases, only 12.5% lead to any sexual offence conviction, and a mere 6.5% end in a conviction for the original charge made by police (Daly & Bouhours, 2010). This pattern of attrition is only observed in sexual assault cases and not in any other type of crime (see Jehle, 2012). One possible explanation for this high attrition and low rate of conviction is that decision‐makers, such as jurors, are influenced by common misperceptions about sexual assault.

### 1.2 Sources of misperceptions about sexual assault

Understanding how misperceptions influence jury decision‐making has important implications, as they influence jurors’ decisions about whether or not to convict (Dinos et al., 2015). Further, police decisions to charge a perpetrator and prosecutor’s decisions to proceed with a case rely partly on their predictions of how a jury may react (Lapsey et al., 2022; Lievore, 2004; Morabito et al., 2019). Research suggests that there are several sources of misperceptions, including rape myths and victim stereotypes. Burt (1980) defined rape myths as “prejudicial, stereotyped, or false beliefs about rape, rape victims, or rapists” that serve to trivialise or justify sexual assault. Rape myths can often be classified into myths about perpetrators, myths about victims, or myths about the offence itself (the latter also referred to in the literature as *event schemas*; Baldwin, 1992). Common myths about perpetrators include that they are simply unable to control their sexual urges or are mentally ill, whereas common myths about victims include that they secretly desire to be raped or only accuse someone of rape because they regret consensual sex (Payne et al., 1999). Further, many people believe that sexual assault happens between strangers in a secluded, public location (Clark & Carroll, 2008; Ryan, 1988) when, in reality, the majority of sexual assaults occur between victims and perpetrators who know each other in a private residence (Australian Bureau of Statistics, 2021; Australian Institute of Health and Welfare, 2020; Cox, 2016). Research suggests that the more jurors endorse these rape myths, the less likely they are to believe that the victim is believable and credible (e.g., Bohner & Schapansky, 2018; Süssenbach, 2016).

Stereotypes about how “genuine” victims behave during and after a sexual assault also impact jurors’ evaluation of the victim. There is a strong expectation that victims will vigorously physically resist the perpetrator, immediately report to police, and display visible distress when recounting the assault (Tidmarsh & Hamilton, 2020). Unsurprisingly, these expectations do not align with reality. Victims are more likely to freeze and cooperate or verbally resist rather than engaging in physical resistance (Larsen et al., 2015; Marx et al., 2008). Similarly, although some victims do show distress when recounting the assault, many victims do not (Burgess & Carretta, 2016; Carretta & Burgess, 2013). This disconnect between inaccurate stereotypes and reality is problematic, as jurors may rely on these stereotypes when evaluating a victim’s evidence. For example, female victims who show distress are consistently perceived to be more credible than those who do not appear distressed, even though emotionality does not predict the truthfulness of a victim’s account (Nitschke et al., 2019).

#### 1.2.1 Gender identity and misperceptions about sexual assault

The content of biases and misperceptions can change depending on the victim’s gender identity. To date, research has largely focussed on similarities and differences in jurors’ beliefs about male and female victims, with little beyond the gender binary. For example, both female and male victims are expected to physically resist the perpetrator and for the perpetrator to have used a weapon to subdue them (Hine et al., 2021; Thelan & Meadows, 2022). However, there are also a range of beliefs that apply to only male or female victims. For example, many people incorrectly believe that women tend to falsely accuse someone of sexual assault after regretting having consensual sex (Thelan & Meadows, 2022). On the other hand, a common misbelief that male victims who have experienced sexual assault perpetrated by a man must be same‐sex attracted or behaved in a way that made the perpetrator believe they were same‐sex attracted (Hine et al., 2021).

Another key difference in misperceptions held about male and female victims relates to their emotional response to sexual assault. As discussed, female victims are expected to be traumatised and emotionally distressed (e.g., Ask, 2010), yet male sexual assault victims are expected to be less traumatised or even able to deal with the assault without help (Hine et al., 2021). These beliefs may be partially informed by gender stereotypes which prescribe the types of emotions that are considered acceptable for men and women to display (Bosma et al., 2018). For example, it is considered more socially acceptable for women to experience and express distress in response to negative life events than it is for men (Fischer et al., 2013). It is also considered less socially acceptable for men to show emotion when stressed (Brody et al., 2016). Given this, there may be reason to believe that the content of misperceptions about transgender victims of sexual assault differs to those about cisgender victims.

To our knowledge, there is a lack of empirical research exploring the content of stereotypes that jurors may hold that are specific to transgender victims of sexual assault (gathered via stereotype elicitation methodology), that could potentially lead to unjust outcomes for transgender victims in sexual assault trials. However, broader research demonstrating frequent negative stereotyping of transgender people suggests that common misperceptions about ‘genuine’ sexual assault victims are likely to disproportionately affect transgender people (Howansky et al., 2019). For example, transgender individuals frequently confront inaccurate stereotypes depicting them as mentally ill, deviant, and sexual which may lead to greater victim‐blaming of transgender victims (Billard, 2018; Howansky et al., 2019). Although it is possible that some individuals called as jurors may be likely to evaluate transgender victims more favourably than other jurors, the research literature on how transgender individuals are perceived suggests that most stereotypes are likely to lead to unfavourable evaluations of transgender victims.

#### 1.2.2 Intervening on misperceptions about sexual assault

Policymakers have begun to act on research that suggests that misperceptions about victims of sexual assault unfairly influence decisions made about victim evidence in criminal trials (e.g., New South Wales Law Reform Commission, 2020). In particular, educational judicial instructions have been introduced in several criminal law jurisdictions. Educational instructions attempt to assist the jury in combatting the influence of stereotypes about victims and sexual assault more broadly in their decision‐making by explaining that common misperceptions about sexual assault victims are incorrect (e.g., Goodman‐Delahunty et al., 2011). Educational instructions have been introduced in the United Kingdom, the jurisdictions of Victoria and New South Wales in Australia, and in Canada.

For example, the United Kingdom has incorporated a suite of educational instructions into the Crown Court Compendium that target “the dangers of assumptions” (Picton et al., 2023). Judges are permitted to give a range of instructions to the jury warning them against using any pre‐existing beliefs about indicators of (un)truthfulness on the victim’s part or of consent being given in sexual assault cases. Judges can explicitly warn jurors that delays in reporting, inconsistencies in the victim’s account, or a lack of emotional distress when giving evidence should not be used as indicators that the victim was being untruthful in their evidence. Judges can select from the list of educational instructions and provide as many of the instructions needed to combat the stereotypes raised by the context of the evidence or advocates’ arguments in a particular case (Picton et al., 2023). Given that misperceptions about sexual assault vary depending on the gender identity of the victim, it is critical to ensure that educational judicial instructions also address differences in misperceptions as a function of the gender identity of the victim.

### 1.3 Current article

Given the high rates of sexual assault experienced by transgender people paired with low levels of reporting and low conviction rates, it is imperative that we find ways to improve case outcomes for transgender victims of sexual assault. One way of doing so may be to improve jurors’ perceptions of transgender victims, however little is known about what misperceptions jurors may hold about transgender victims and how these misperceptions affect their decision‐making. Further, it is unclear what legal interventions (e.g., judicial instructions) are currently available to protect against potential prejudice against transgender victims. Therefore, the first aim of this article was to examine the existing empirical research on jurors’ perceptions and decision‐making in cases involving transgender victims of sexual assault through a literature review. The second aim of this article was to conduct a policy review of judicial instruction repositories to determine what, if any, instructions are given to juries about gender identity in sexual assault cases.

## 2 LITERATURE REVIEW OF EMPIRICAL RESEARCH

### 2.1 Method

#### 2.1.1 Search strategy

The purpose of this literature review was to examine the current state of the literature on (mock) jurors’ perceptions of transgender victims of sexual assault. This literature review was not intended to be a systematic review, but rather to explore the current state of the literature on perceptions of transgender victims. We consulted an experienced law librarian to develop the search strategy for the literature review using a problem‐analysis matrix to derive keywords from the research question and undertook scoping searches of EBSCOhost, Westlaw and ProQuest. ProQuest was selected as the database for this review as the search returns contained less material which was irrelevant to the purpose of our review. We searched ProQuest on 8 September 2023 using the following search strategy: (transgender OR transsexual OR transsexual OR “gender variant” OR “gender non‐conforming” OR “gender identity”) AND (“sexual assault” or rape) AND (jury OR juror) AND (victim OR complainant) AND (guilt OR blame OR verdict) which identified a total of 326 records. We also searched the reference lists of eligible studies to identify any additional studies that met our inclusion criteria. To be eligible for inclusion in the narrative literature review, studies had to be: (1) an experimental study (2) reported in English, (3) published in a peer‐reviewed publication, (4) about a criminal case involving sexual assault, (5) included at least one condition in which the victim’s gender identity is transgender, and (6) participants in the study made judgments about perceptions of the victim, the offence, or decisions about case outcomes (e.g., verdict). Title and abstract screening of all articles was conducted independently by both authors using the Covidence online literature review management tool which identified three relevant studies. After full text screening conducted independently by both authors, all three studies were retained in the narrative review. One additional study was identified through searching the reference lists of these studies, resulting in a total of four studies included in the narrative review.

### 2.2 Results

To avoid any confusion, in this literature review we generally use the gender and gender identity terminology used by the original authors (e.g. “transgender female” rather than “transgender woman”).

#### 2.2.1 Study characteristics

A total of four experimental studies have investigated jurors’ perceptions of sexual assault victims who identify as transgender (Carter et al., 2023; Davies & Hudson, 2011; Ellingwood et al., 2023; Miller & London, 2023). There was a total of 1099 participants (63.1% women, 36.9% men) across all four studies. Two studies (50%) excluded participants based on their gender identity or sexual orientation, with one study excluding participants who identified as transgender, and another excluding participants who identified as transgender, gay, bisexual, or other sexual orientation. In two studies, participants received course credit in exchange for their participation, however in the remaining two studies it was unclear whether (or how) participants were remunerated for their participation. In all four studies, all transgender victims identified within the gender binary (i.e., a boy/man or girl/woman), thus the studies did not include transgender victims who identify as non‐binary, gender‐fluid, or another term within their experimental design. Further information about the included studies including mode of participation, study design, case stimuli, offence type, relationship of the perpetrator to the victim, perpetrator gender, and dependent variables can be found in Table 1 below.

**TABLE 1:** Characteristics of all studies included in the literature review.

Study	Participants	Mode of participation	Case stimuli	Study design	Offence‐type	Relationship of perpetrator to the victim	Perpetrator gender	Dependent variables
Carter et al. (2023)	223 (61.4% women, 38.6% men) students from a liberal arts college in the USA	In person	Trial transcript	2 (participant gender: Female, male) x 4 (victim gender identity: Cisgender female, cisgender male, transgender female, transgender male) x 2 (judicial instructions: Present, absent) between‐subjects factorial design	Rape	Stranger	Male perpetrator	Victim blame
								Crime severity
								Commission (how likely they thought the defendant committed the crime)
								Verdict
								Confidence in verdict decision
								Sentence length (if a guilty verdict)
Davies and Hudson (2011)	133 (43.6% women, 56.4% men) community members from Liverpool, England	In person	Case summary	5 (victim gender identity/sexual orientation: Heterosexual male, homosexual male, male crossdresser, transgender male, transgender female) x 2 (participant sexual orientation: Heterosexual, homosexual) x 2 (participant gender: Female, male) between‐subjects factorial design	Rape	Stranger	Male perpetrator	Victim blame
								Crime severity
Ellingwood et al. (2023)	375 (73.8% women, 26.2% men) undergraduate students from a Canadian university	Online	Trial transcript	4 (victim gender identity: Cisgender female, cisgender male, transgender female, transgender male) x 3 (victim sexual orientation: Heterosexual, homosexual, or bisexual) between‐subjects factorial design	Aggravated sexual assault	Stranger	Male perpetrator	Perceptions of the victim (credibility, believability, honesty, accuracy, reliability)
								Perceptions of the defendant (credibility, believability, honesty, accuracy, reliability)
								Guilt
								Verdict
Miller and London (2023)	368 (60% women, 40% men) community members ‐ U.S. citizens recruited from Amazon Mechanical Turk	Online	Case summary	2 (victim gender: Girl, boy) x 2 (victim gender identity: Cisgender, transgender) x 2 (victim sexual orientation: Heterosexual, homosexual)	Child sexual abuse (single incident)	Teacher	Male perpetrator	Victim believability
								Defendant believability
								Victim responsibility
								Defendant responsibility
								Belief that sexual contact had occurred
								Guilt
								Verdict

#### 2.2.2 Results of individual studies

In this section, we focus solely on the effects relating to the victim’s gender identity for each study. Carter et al. (2023) investigated the effects of victim gender identity, participant gender, and judicial instructions to jurors to ignore victim gender identity on victim blame, crime severity, likelihood that the defendant committed the crime, verdict, and sentencing recommendations in a case involving stranger rape. The authors manipulated the gender identity of the victim as either: a cisgender female, cisgender male, transgender female, or transgender male. Furthermore, half of the participants received instructions from the judge at the beginning of the trial to ignore the gender identity of the victim while the remaining participants did not receive these instructions. As our article also explores the use of judicial instructions relating to gender identity, we also include discussion of these results. There was a significant main effect of victim gender identity on crime severity ratings, with the crime committed against the cisgender female victim considered more severe than against the transgender male victim. Further, the crime was considered more severe when committed against a transgender female victim than a transgender male victim. However, judicial instructions had no effect on crime severity and neither victim gender identity nor judicial instructions influenced victim blame, likelihood that the defendant committed the crime, verdict, or sentencing decisions.

Davies and Hudson (2011) investigated the effects of victim gender identity and sexual orientation (grouped together), participant gender, and participant sexual orientation on victim blame and perceived crime severity in a stranger rape scenario. The authors manipulated whether the victim identified as a heterosexual male, homosexual male, male crossdresser, transgender male, or transgender female. Results revealed a significant main effect of victim gender identity/sexual orientation on victim blame, but only between the two conditions with the largest difference in scores. Specifically, participants tended to blame the male crossdressing victim more than the heterosexual male victim. In contrast, there was no effect of victim gender identity/sexual orientation on crime severity ratings.

Ellingwood et al. (2023) investigated the effects of victim gender identity and victim sexual orientation on perceptions (including credibility, believability, honesty, accuracy, and reliability) of both the victim and the defendant as well as judgments about guilt (dichotomous and continuous) in a case of aggravated sexual assault. The authors manipulated the victim’s gender as either a cisgender female, cisgender male, transgender female or transgender male, and manipulated the victim’s sexual orientation as either heterosexual, homosexual, or bisexual. The authors found that participants were more likely to render a guilty verdict when the victim was a cisgender male or female victim compared to a transgender male victim. However, there was no effect of victim gender on perceptions of the victim, perceptions of the defendant, or continuous ratings of guilt.

While Davies and Hudson (2011), Carter et al. (2023), and Ellingwood et al. (2023) all investigated perceptions of transgender adult victims, Miller and London (2023) investigated perceptions of transgender child sexual abuse victims. They examined the effects of victim gender (boy or girl), victim gender identity (cisgender or transgender), and victim sexual orientation (heterosexual or homosexual) on dichotomous and continuous guilt judgments, belief that sexual contact had occurred, and credibility and responsibility of both the victim and the defendant. The case involved alleged sexual abuse of a 15‐year‐old by their 34‐year‐old male teacher. Results revealed a significant main effect of victim gender identity on victim credibility ratings, with cisgender victims perceived to be more credible than transgender victims. Similarly, there was a significant main effect of victim gender identity on defendant credibility ratings, with the defendant perceived to be more credible when the victim was transgender compared to cisgender. Victim gender identity also had an effect on participants’ verdicts, with participants more likely to reach a guilty verdict when the victim was cisgender compared to transgender. However, victim gender identity did not have an effect on belief that sexual contact had occurred, victim responsibility, defendant responsibility, or likelihood of guilt.

Individually these studies provide some support for differences in perceptions and judgments about sexual assault cases that involve transgender victims versus cisgender victims, potentially disadvantaging transgender victims in the criminal justice system. However, results were inconsistent across dependent variables and studies. For example, only 50% of studies found a significant difference in victim blame attributed to cisgender versus transgender victims, with transgender victims more likely to be blamed for their victimisation. Similarly, of the three studies measuring perceived crime severity, only one study found a significant difference between cisgender and transgender victims, with the crime committed against the transgender victim considered to be less severe. Further, two of the three studies measuring participants’ verdict preference found a significant difference between cases involving transgender versus cisgender victims, with cases involving cisgender victims resulting in a higher percentage of guilty verdicts but no significant differences on continuous measures of defendant guilt. Interestingly, the study involving adult victims found that participants were more likely to reach a guilty verdict when the victim was a transgender female compared to male, while the study involving child victims found that participants were more likely to reach a guilty verdict when the victim was a transgender boy compared to girl. Methodological limitations may explain some of the varying findings in these studies, which we discuss in depth in the discussion.

## 3 POLICY REVIEW

### 3.1 Method

#### 3.1.1 Inclusion parameters

The purpose of this policy review was to understand the instructions the jury are given by the judge about the victim’s gender identity in a criminal trial for sexual assault. For this review, our focus was on judicial instructions given in criminal trials for sexual assault in five common law western countries: the United Kingdom, USA, Canada, New Zealand and Australia. This included instruction repositories for all states in Australia and the USA and all countries in the United Kingdom. In the USA, we also searched federal criminal law instruction repository in Lexis+ (which includes pattern instructions for the First, Third, Fifth, Sixth, Seventh, Eighth, Nineth, Tenth and Eleventh Federal Circuits). We included a criminal law jurisdiction in the review when we were able to access the repository of judicial instructions publicly online or via commercial collections of instructions.

#### 3.1.2 Search strategy

We consulted with an experienced law librarian on the search strategy to identify repositories of judicial instructions. In consultation with the law librarian, we identified that there was no appropriate academic database to use to identify repositories of judicial instructions. Repositories for judicial instructions are commonly hosted on a website maintained by the courts organisation in that jurisdiction. As a result, we searched the Internet for these websites. We searched for repositories of instructions using combinations of the following terms: jurisdiction name (e.g., Canada or New South Wales) AND criminal trial AND (instruction OR direction OR benchbook). After searching for instruction repositories on courts websites, we also used two commercial databases of American instruction repositories (via the Secondary Source collection in Westlaw and the US Instruction Collection in Lexis+) to gain access to additional instruction repositories.

#### 3.1.3 Data extraction and coding

To extract relevant instructions from repositories of instructions, we searched each repository for mentions of the word ‘gender’. For each repository of instructions, we coded whether there were judicial instructions that included the word gender. For repositories where there were instructions that mentioned gender, we extracted information about the instructions that were specific to sexual violence or how witnesses should be evaluated. We extracted the following information about each instruction: the title of the instruction, a brief description of the instruction, and the text of the instruction. After we extracted information about each instruction, we grouped instructions into categories based on their content. A full list of instructions included in the review is available on the Open Science Framework: https://osf.io/xgtb6/

### 3.2 Results

#### 3.2.1 Availability of instruction repositories

We were not able to access instruction repositories for all criminal law jurisdictions in Australia, New Zealand, and the USA. Table 2 shows the percentage of instruction repositories found in each country and lists the criminal law jurisdictions where we were unable to access all instruction repositories (e.g., some states in Australia and the USA do not make their repositories available).

**TABLE 2:** Number and percentage of instructions mentioning gender identity in each category.

Country	Total criminal law jurisdictions	Percentage (and count) of criminal law jurisdictions with accessible instruction repositories	Criminal law jurisdictions without accessible instruction repositories
Australia	8	50% (4)	Western Australia, Northern Territory, Australian Capital Territory, Tasmania
Canada	1	100% (1)	‐
New Zealand	1	100% (1)	‐
United Kingdom	4	75% (3)	Republic of Ireland
United States of America	52	86.5% (45)	Idaho, Missouri, Nevada, New Hampshire, South Dakota, Texas

Of the instruction repositories that we were able to access for the review, not all criminal law jurisdictions had instructions about how gender identity that could be used in a criminal trial for sexual violence. This included general instructions (which can also be used in other criminal trials) and those designed for sexual violence trials. Less than half of the jurisdictions (40.7% of jurisdictions with an instruction repository we were able to access for the review) had instructions about how juries should interpret information about gender identity (including some jurisdictions in Australia, the United Kingdom, the United States and Canada).

#### 3.2.2 Types of instructions

In total, we identified 31 judicial instructions that mentioned gender or gender identity across the instruction repositories that we were able to access. We grouped these instructions into nine different categories based on their content. These categories and the number and percentage of instructions that fell into each category are provided in Table 3.

**TABLE 3:** Number, percentage and jurisdiction of instructions mentioning gender identity in each category.

Instruction‐type	Frequency	Percentage	Jurisdictions
Cross‐examination	1	3.2%	New South Wales
Gender identity warning	2	6.5%	Canada, Colorado
Gendered violence case	4	12.9%	England, Wales and Victoria
Offence	4	12.9%	Scotland, Queensland and New South Wales
Opening instructions	17	54.8%	Canada, Arizona, California, Colorado, Connecticut, Hawaii, Illinois, Minnesota, New York, North Dakota, Oregon, Pennsylvania, Rhode Island, West Virginia and Wisconsin
Witness credibility	3	9.7%	Michigan and Wisconsin
Total	31	100.0%	‐

##### Opening instructions

The most common type of instruction that referenced gender or gender identity was opening instructions. Opening instructions are instructions that judges give to the jury at the start of the trial and these instructions are given in all criminal trials of any offence type. Typically, these instructions are designed to introduce the jury to how the trial will work, what the different roles of the people in the courtroom are, and to explain to the jury what their task will be in the trial. In opening instructions, gender or gender identity was typically mentioned in instructions about the jury’s role in the trial. Typically, gender or gender identity was mentioned in instructions that warned the jury against prejudice in their decision‐making. For example, in Hawaii, the following instruction is given as part of the opening instructions: “You must not let bias, sympathy, prejudice, or public opinion influence your decision. You must not be biased in favour of or against any party or witness because of the person’s actual or perceived race, colour, ancestry, national origin, ethnicity, sex, gender, gender identity, sexual orientation, marital status, age, disability, religion, socioeconomic status, or political affiliation.”

##### Sexual offences

Some instructions about sexual offences also mentioned gender or gender identity. People with transgender identities were specifically mentioned in instructions for a small number of criminal offences including illegal distribution of intimate images or offences about sexual touching without consent. In these instructions, transgender people were specifically identified as a group of people who could be victims of these crimes. Transgender identity was also mentioned as an aggravating factor for crimes in one jurisdiction if the motivation for criminal offending was the victim’s transgender identity.

##### Gendered violence cases

A few jurisdictions also had instructions that mentioned gender identity as part of instructions developed specifically for sexual and domestic violence cases. For example, in the Australian jurisdiction of Victoria, there is an instruction that can be given in cases involving sexual assault offences to educate the jury about the wide range of circumstances in which non‐consensual sexual interactions can occur. Juries in Victoria can be specifically warned that “⋯ sexual acts can occur without consent between all sorts of people including … people of any gender identity, including people whose gender identity does not correspond to their designated sex at birth.” A similarly broad warning about gender identity in sexual assault cases can also be given in the jurisdiction of Colorado (USA). In the United Kingdom, a warning can be given to the jury about the gender identity of people who experience domestic violence: “There is no such thing as a typical victim of domestic abuse and no such thing as a typical abuser; domestic abuse can occur irrespective of age, gender, and social circumstances.”

##### Witnesses

Most repositories of judicial instructions contain instructions to help guide the jury in how they should evaluate the evidence given by witnesses. Two jurisdictions had general witness instructions that mentioned gender identity within their instructions about how witnesses should be evaluated generally (Michigan and Wisconsin, USA). Both general witness instructions warn jurors that they should not allow their evaluation of witnesses in the case to be prejudiced by the gender identity of the witness. Although not an explicit instruction delivered to jurors, the benchbook in Victoria (Australia) specifically prohibits judges, prosecutors, and defence lawyers in sexual assault trials from saying anything that states or implies that victims with a specific gender identity are less credible or require more careful scrutiny by the jury.

##### Gender identity warnings

A small number of jurisdictions have instructions that can be given to the jury if gender identity of a witness is raised in a criminal trial (in Canada and Colorado, USA). For example, in Colorado, when gender identity is raised in the evidence for a criminal case judges can instruct the jury: “You are about to hear evidence relating to [a victim’s]/[the defendant’s]/[a witness’s] actual or perceived gender identity, gender expression, or sexual orientation. You are instructed that you must not allow bias or any kind of prejudice based upon gender identity, gender expression, or sexual orientation to influence your decision.”

##### Cross‐examination

Although not necessarily an instruction given to the jury, in New South Wales, judges are empowered to intervene to prevent a witness from answering any question in cross‐examination which is improper. In the benchbook, judges are required to consider the witnesses’ personal characteristics, including gender, in evaluating whether a question posed by counsel is improper in cross‐examination.

## 4 DISCUSSION

In this research we had two main aims. First, we aimed to examine published empirical studies on jurors’ perceptions of transgender sexual assault victims through a literature review. Second, we conducted a policy review of available judicial instruction repositories to determine the types of instructions juries might be given about gender identity in sexual assault cases.

### 4.1 Perceptions of transgender sexual assault victims

In the literature review of empirical studies, we identified four studies that investigated (mock) jurors’ perceptions and decision‐making in cases involving transgender victims of sexual assault. The collective findings from these studies revealed mixed results regarding the influence of victim gender identity on juror perceptions and decision‐making. Although some studies fund significant differences in victim blame, crime severity, and verdicts of sexual assaults committed against transgender compared to cisgender victims, others found no such differences. However, when significant differences were observed they were more favourable towards cisgender victims, highlighting potential biases that may disadvantage transgender victims within the criminal justice system. Methodological limitations may explain some of the inconsistencies observed between variables and studies.

#### 4.1.1 Potential limitations regarding statistical power

Firstly, the inconsistent findings may be due to issues of statistical power (i.e., the probability that the study will detect a significant effect if one exists, O’Keefe, 2007). When research has low statistical power, the likelihood of obtaining a Type II error (i.e., a false negative) increases (Lakens & Caldwell, 2021). However, low statistical power also decreases the likelihood that a statistically significant effect is a true positive and inflates the effect size (Button et al., 2013; Ioannidis, 2005). Generally, underpowered research has been a consistent issue in psychology. Cohen (1962) calculated that the average power in psychological research was 18% for small effects, 48% for medium effects, and 83% for large effects. More than half a century later there seems to have been no improvement, with the average power estimated at 23% for small effects, 60% for medium effects, and 78% for large effects (Szucs & Ioannidis, 2017).

Research has shown that only 2.9% of psychology articles report a priori power analyses to inform their sample size (Tressoldi & Giofré, 2015). Importantly, none of the studies included in the literature review reported that a power analysis had been conducted. One study (Carter et al., 2023) reported that their sample size was sufficient to detect large effect sizes but did not describe a priori power analyses undertaken to ensure this or which effect they were referring to (i.e., a main effect or interaction). In this study, the authors hypothesised an attenuated (i.e., moderated) three‐way interaction. Although it is difficult to conduct power analyses to determine sample size for interactions (Sommet et al., 2023), the sample size required to detect a large three‐way interaction is likely larger than that used in this study (Giner‐Sorolla, 2018). Given the between‐subjects nature of the experimental designs with multiple factors and that typical effect sizes in social psychology research are small‐to‐medium (Richard et al., 2003), it is very possible that the studies included in the literature review were underpowered, leading to the observed inconsistencies in results. In future research, we recommend that authors conduct power analyses, assuming small to moderate effects will be observed, and report enough detail about the power analyses such that the analyses can be replicated.

#### 4.1.2 Limitations regarding the ecological validity of scenarios

In addition to the potential statistical power issues, the existing literature also lacks ecological validity in terms of the type of sexual assault cases used as stimuli. All studies that investigated perceptions of adult victims of sexual assault (Carter et al., 2023; Davies & Hudson, 2011; Ellingwood et al., 2023) employed a stereotypical stranger sexual assault in an outdoor public location involving some form of physical harm. However, in reality, the majority of sexual assaults are perpetrated by someone known to the victim, in a residential location, and without physical injury (Australian Bureau of Statistics, 2021; Australian Institute of Health and Welfare, 2020; Waterhouse et al., 2016). Therefore, the scenarios used in these studies are not representative of the most common types of sexual assault. Although research on cisgender victims has shown that more stereotypical cases elicit less victim blame than less stereotypical cases (e.g., Koepke et al., 2014; Masser et al., 2010; Persson & Dhingra, 2022; Romero‐Sánchez et al., 2018; Schuller et al., 2010), it is unclear whether this effect is more or less pronounced for transgender victims. Therefore, future research on perceptions of transgender victims of sexual assault should consider using scenarios that reflect the most common forms of sexual assault, including known perpetrators, lack of resistance or physical injury, and delayed reporting.

Further, future research should consider how multiple marginalised identities interact when evaluating transgender victims of sexual assault. Intersectionality theory proposes that having multiple marginalised identities can compound negative treatment from others (Crenshaw, 1990). Indeed, having multiple marginalised identities (e.g., identifying as both transgender and a person of colour) increase one’s risk of sexual assault victimisation (Coulter et al., 2017; Coulter & Rankin, 2017; Grant et al., 2011) and could reduce one’s willingness to report to police (Donne et al., 2018). For example, Staples and Fuller (2021) surveyed transgender adults in the USA about sexual assault experiences based on their race and perceived transgender visibility. For individuals who reported low transgender visibility, sexual assault severity was similar regardless of race. However, among those who reported high transgender visibility, people of colour reported higher sexual assault severity than white individuals. This clearly highlights the role of intersectionality in sexual assault experiences, though substantially more research is needed to understand how multiple marginalised identities interact to affect perceptions of the evidence given by transgender sexual assault victims.

#### 4.1.3 Excluding gender and sexually diverse participants

We found that 50% of the studies included in the literature review explicitly excluded data from gender and sexually diverse participants. While this was likely done for methodological reasons rather than any ill intent, it is important to recognise that such exclusions may unintentionally contribute to the underrepresentation and marginalisation of gender and sexually diverse individuals (Cameron & Stinson, 2019). One reason why researchers may exclude data from gender or sexual diverse participants is because they planned to include gender or sexuality as factors in their analyses but did not reach a suitable sample size to make robust inferences. However, this runs the risk of research only being able to make inferences about the gender binary. Therefore, researchers could either ensure they adequately sample gender and sexually diverse participants rather than relying on a convenience sample or consider whether gender is simply being used as a proxy variable. Regarding the latter, Hyde et al. (2019) argue that, in many circumstances, gender could be replaced by other variables that tend to correlate with particular gender or sex categories. In the case of research on sexual assault, it is worth considering whether we are indeed interested in the effect of participant gender on perceptions of victims and decision‐making outcomes, or whether variables that tend to cluster around particular gender identities (e.g., rape myth acceptance) serve a better function and allow for greater participant inclusivity.

Further, how researchers ask participants about their gender has been shown to be problematic in and of itself. A review of 106 empirical studies published in the journal *Psychological Science* found that 76% used binary measures of gender that do not accurately reflect non‐binary and some transgender individuals (Cameron & Stinson, 2019). The authors advocate for the use of blank textboxes for participants to self‐identify gender in their own terms and provide open‐access code to categorise these open‐ended responses. Alternatively, if relevant to the research question, researchers may wish to adopt a two‐step approach, asking about participants’ gender identity and sex assigned at birth. Carefully considering participant exclusion criteria and adopting more inclusive approaches to measuring participant gender can help to ensure that research respects and reflects gender diversity.

### 4.2 Judicial instructions about gender identity

In the policy review of judicial instructions, we identified 33 instances of instructions given to the jury by the judge about the gender identity of victim witnesses in sexual assault trials. There are three key findings of our review. First, around half of the instruction repositories that we were able to access contained instructions for the jury about using information about the gender identity of witnesses. As we were not able to access the instruction repositories for all criminal law jurisdictions, it is possible that instructions about gender identity are only in the instruction repositories for a minority of criminal law jurisdictions in our five countries of interest. Second, most instructions to the jury about gender identity were part of opening instructions. On the one hand, it is positive to see opening instructions that include a warning to jurors that they should not use gender identity to unfairly make decisions about witnesses and other participants in criminal trials. However, these general warnings may not be substantial enough to protect transgender victims from jurors unfairly using gender identity to make decisions in sexual assault cases. Third, only a handful of jurisdictions have developed specialist educational instructions about not using gender identity unfairly in cases of sexual assault or domestic violence (which can include sexual assault charges). It may be necessary for more jurisdictions to implement these specialist educational instructions to ensure that jury decisions in sexual assault cases are not unfairly affected by inaccurate stereotypes about transgender victims. However, as research suggests that instructions can have varied effects on jury decision‐making, it is important to consider whether these instructions are likely to be effective in helping jurors to make accurate and unbiased decisions.

#### 4.2.1 Instructions may not be effective

Most of the instructions about gender identity we identified in the policy review used one of two strategies to provide the jury with information about how gender identity was to be considered in their decision‐making. The first strategy used was to warn the jury that they should not allow their decisions to be affected or biased by the gender identity of any participant in the trial. For example, most opening instructions — the most frequent type of instruction to mention gender identity — explicitly warned jurors about using the gender identity of any trial participant to make decisions about the case. The second strategy used was to inform jurors that beliefs they may have about people with diverse gender identities may be incorrect. For example, some of the specific instructions developed for gendered violence cases focussed on explaining that people of any gender identity could be a victim of these types of crimes.

Judicial instructions are intended to assist jurors in making decisions in criminal trials. Unfortunately, much of the empirical literature suggests that judicial instructions have mixed effects on jury decisions (e.g., Bornstein & Greene, 2017; Devine, 2012). Sometimes judicial instructions help jurors make less biased decisions (e.g., Goodman‐Delahunty et al., 2011), whereas other times they do not (e.g., Nitschke et al., 2023). To our knowledge, only one study to date — included in the literature review — has specifically investigated if instructions about gender identity assist jurors in making accurate decisions about the evidence given by gender diverse victims (Carter et al., 2023). The instruction provided by the judge in this study was an opening instruction, warning jurors that the victim’s gender should not be focussed on when deciding the verdict. However, we cannot draw any conclusions about the effectiveness of the instruction, given the instruction did not have any effect on countering a bias against transgender victims when participants decisions showed a gender bias and only had an effect on participants’ ratings of defendant culpability when participants did not show a gender bias in their decisions. Further, potential issues with statistical power means that we are unable to determine whether any non‐significant effects of judicial instructions were due to a Type II error (i.e., false negative) or because the instruction was ineffective.

More generally, instructions that warn jurors not to be influenced by the gender identity of a witness are similar to curative instructions, which tell jurors to ignore bias and other information that they are not allowed to use to inform their decision‐making (e.g., pre‐trial publicity of the case). A larger body of research has investigated the effectiveness of curative instructions, finding that jurors have difficulty following these instructions in their decision‐making (e.g., Daftary‐Kapur & Penrod, 2018; Lynch et al., 2022). For example, in a meta‐analytic review of curative instructions about inadmissible evidence, jurors’ decisions about the trial were influenced by inadmissible evidence even when they received curative instructions explicitly telling them not to use this evidence (Steblay et al., 2006). Further, the meta‐analysis found that the instructions may have the opposite effect, causing jurors to attribute *greater* importance to the evidence than if the instruction was not given.

As this research suggests that judicial instructions do not always function as intended, it is critical that further research explores the efficacy of instructions about gender identity, including warnings and instructions identified in this review, to adequately protect against prejudicial decisions being made against transgender victims in sexual assault trials. In future mock jury studies, researchers should concurrently manipulate case evidence strength, complainant gender identity and type of judicial instruction (with a no instruction control level). This‐type of design would enable researchers to understand whether judicial instructions remove effect of gender identity on case decisions and/or sensitise jurors to case evidence (suggesting that decision‐making is becoming more accurate). This would help to advance our understanding of how instructions may or may not influence jury decisions in these cases. Future research could also investigate the effectiveness of other legal safeguards, including expert evidence which shows some promising effects on jury decisions (Cossins, 2013). For example, Brekke and Borgida (1988) found that mock jurors found a rape victim more credible, were less likely to believe that she consented, and were more likely to convict the defendant when an expert presented evidence to counter misconceptions about rape than when no expert evidence was presented. Importantly, research has shown that both the timing of the expert testimony (i.e., delivered prior to the victim’s testimony) and specificity of the testimony (i.e., directly linking the empirical research to the specific case) have an impact on the effectiveness of the expert evidence (Brekke & Borgida, 1988; Gabora et al., 1993; Goodman‐Delahunty et al., 2021). However, for expert evidence to be effective in countering misconceptions and stereotypes about transgender victims, it is first necessary to understand what those misconceptions and stereotypes are.

## 5 CONCLUSION

Transgender victims of sexual assault face disproportionately higher rates of victimisation than cisgender victims (Flores et al., 2021), yet have some of the lowest reporting rates among all victims (Langenderfer‐Magruder et al., 2016). High attrition of sexual assault cases throughout the criminal justice system and low conviction rates may be due to common misperceptions that decision‐makers, such as jurors, have about sexual assault (Cox., 2016; Lonsway & Archambault, 2012). However, little is known about how, or even whether, jurors are influenced by these common misperceptions when evaluating sexual assault cases involving transgender victims. Further, it is unclear whether there are effective legal safeguards, such as judicial instructions, to guard against these misperceptions influencing jurors’ decisions. In this article, we found only four published empirical studies to date that have examined jurors’ perceptions and decisions in cases involving transgender victims compared to cisgender victims (Carter et al., 2023; Davies & Hudson, 2011; Ellingwood et al., 2023; Miller & London, 2023). Overall, these studies provide some support that transgender victims may be evaluated more negatively, but findings were mixed between dependent variables and studies and studies had several methodological limitations. Given that transgender individuals face disproportionately higher rates of victimisation, it is clear that more research is needed to gain a better understanding of how transgender victims may be evaluated in the criminal justice system, the biases and misconceptions that may lead to negative evaluations, and how the criminal justice system can better ensure equitable justice for all victims, regardless of gender identity. We also found that many jurisdictions allow judges to provide instructions to the jury about gender identity. The majority of these instructions (53.6%) were opening instructions provided at the beginning of the trial that warn jurors against prejudice in their decision‐making. However, research has shown mixed effects with regards to the effectiveness of judicial instructions (e.g., Bornstein & Greene, 2017; Devine, 2012). In conclusion, more research is urgently needed to investigate specific stereotypes that jurors may have about transgender victims and to evaluate potential legal safeguards to prevent inaccurate stereotypes from influencing their perceptions of victims and ultimate verdict.

## CONFLICT OF INTEREST STATEMENT

The authors declare no conflicts of interest.

## ENDNOTES

^1^
We acknowledge that there are various definitions of sexual assault depending on legal jurisdiction. In this article, we are referring to sexual contact of a person without their consent (that must be freely and voluntarily given). Sexual assault can include rape (forced sexual penetration of a penis, body part, or object into a vulva, vagina, or anus without consent that is freely and voluntarily given).

^2^
We have adopted the American Psychological Association’s (2022) definition, which refers to an individual whose gender identity or expression does not conform to what society typically expects of that individual’s sex assigned at birth. A transgender individual might identify as a man, woman, non‐binary, gender‐fluid, or another term.

^3^
As this article focuses on sexual assault specifically within the context of legal proceedings where the term ‘victim’ is used, we have chosen to use this term throughout. However, we recognise that individuals who have experienced sexual assault may personally prefer alternate terms like ‘survivor’, ‘victim‐survivor’, or others based on their lived experiences.
